# Case report: Enhancing prognosis in severe COVID-19 through human herpes virus coinfection treatment strategies

**DOI:** 10.3389/fcimb.2023.1320933

**Published:** 2024-01-10

**Authors:** Ye Lu, Cuihong Wang, Yuan Wang, Yu Chen, Li Zhao, Yu Li

**Affiliations:** Department of Pulmonary and Critical Care Medicine, Shengjing Hospital of China Medical University, Shenyang, Liaoning, China

**Keywords:** COVID-19, human herpes virus co-infection, case report, glucocorticoids, antiviral therapy

## Abstract

**Background:**

In the context of increasing reports of co-infection with coronavirus disease 2019 (COVID-19), particularly with human herpes viruses (HHVs), it is important to consider the appropriate treatment options for HHVs that have been reactivated by COVID-19.

**Case presentation:**

This study presents two cases of severe COVID-19 with HHV co-infection. The first case involved a critically ill patient with COVID-19 co-infected with herpes simplex virus type 1, confirmed using metagenomic next-generation sequencing, and another patient with severe COVID-19 experiencing Epstein-Barr virus (EBV) reactivation, as evidenced by elevated EBV-DNA levels in the serum. Treatment included high-dose glucocorticoids and sivelestat sodium, with notable improvements observed after initiating ganciclovir anti-herpesvirus therapy.

**Conclusion:**

This study underscores the significance of recognizing HHV co-infections in severe COVID-19 cases and highlights the potential of combining anti-HHV treatment, increased glucocorticoid dosages, and anti-cytokine storm therapy to enhance prognosis.

## 1 Introduction

Coronavirus disease 2019 (COVID-19), caused by the severe acute respiratory syndrome coronavirus (SARS-CoV-2) infection, rapidly and disastrously disseminated worldwide for more than 3 years. COVID-19 represents a significant public health crisis that exerted a substantial impact on global economic growth and disrupted daily life (Dong et al., 2023). As of July 27, 2023, the World Health Organization (WHO) reported that COVID-19 has afflicted approximately 768 million individuals and claimed the lives of over 6.9 million people across 226 countries (Organization, 2021). With the help of vaccines and the early utilization of antiviral medications, mortality resulting from COVID-19 has dropped dramatically (Gao et al., 2023). However, COVID-19 continues to pose a significant threat to vulnerable populations. Consequently, identifying the risk factors for COVID-19 and initiating targeted interventions to enhance the prognosis are of paramount importance for medical research.

Numerous studies have demonstrated that human herpes virus (HHV) detection rates are higher in patients with COVID-19 than in non-COVID-19-infected individuals (Banko et al., 2023). Therefore, HHVs may play a role in exacerbating COVID-19. Until now, nine HHV species have been identified, including herpes simplex virus type 1 (HSV1), herpes simplex virus type 2 (HSV2), varicella zoster virus (VZV), Epstein–Barr virus (EBV), cytomegalovirus (CMV), human beta herpesviruses 6A, 6B, and 7 (HHV-6A, HHV-6B, and HHV-7), and human gama herpesvirus 8 (HHV-8) (Banko et al., 2023). HHVs cause latent infections in approximately 90% of the population (Zubchenko et al., 2022; Peluso et al., 2023), and their activation may lead to a series of adverse outcomes, especially in immunocompromised patients (Carneiro et al., 2022). However, there is a lack of consensus regarding the potential exacerbation of COVID-19 by HHVs as well as the appropriate treatment strategies for the reactivation of HHVs induced by COVID-19.

In this study, we describe two cases of severe COVID-19 with HHV co-infection. We achieved successful treatment outcomes in these two cases. This case report serves as an evaluative study, shedding light on the treatment modalities used in these cases. The objective of this report is to raise awareness of this phenomenon and contribute to the enhancement of clinical outcomes for individuals with severe COVID-19.

## 2 Case description and diagnostic assessment

### 2.1 Case 1

**Figure 1:**
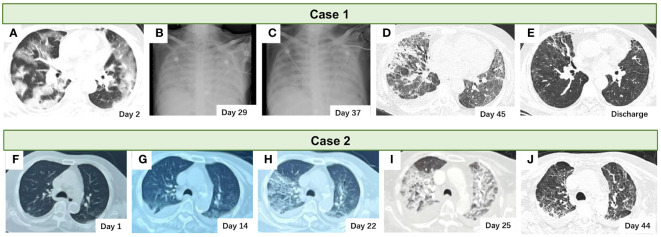
The pulmonary radiological images of the two cases. **(A–E)** The chest CT scan and the X-ray of the case 1 patient. **(F–J)** The chest CT scan of the case 2 patient.

A 64-year-old Chinese man with a medical history of hypertension and hepatocellular carcinoma treated with Lenvatinib presented to our department with fever, dyspnea, and bloody sputum. Six days before admission, the patient had a fever; his maximum body temperature was 39°C. Four days later, the patient experienced dyspnea, cough, and bloody sputum. A physical examination upon admission revealed an urticarial rash all over the patient’s skin and an increased respiratory rate (24 beats/min). A nasopharyngeal swab-PCR tested positive for SARS-CoV-2 RNA. Laboratory examination (**Supplementary Table 1**) at admission revealed type I respiratory failure (PaO_2_:60 mmHg with 15 L/min mask oxygen therapy), hypokalemia (3.2 mmol/L), lymphopenia (0.6×10^9/L), thrombocytopenia (91×10^9/L), myocardial injury (high-sensitivity troponin I:0.0671 μg/L), elevated C-reactive protein (134.4 mg/L), elevated lactate dehydrogenase (824 IU/mL), slightly elevated interleukin-6 (7.16 pg/mL), elevated ferritin (1275.2 ng/mL), and elevated D-dimer (622 µg/L). A chest CT scan revealed multiple bilateral pulmonary ground-glass opacities (**Figure 1A**).

Based on these findings, the patient was diagnosed with severe COVID-19 and treated with nirmatrelvir/ritonavir, methylprednisolone (80 mg, administered every 12 hours via intravenous drip), low molecular heparin calcium (0.4 ml, administered daily via subcutaneous injection), a six-day course of broad-spectrum antibiotic combination of piperacillin sodium and tazobactam sodium (4.5 g, administered every eight hours via intravenous drip), and levofloxacin (0.5 g, administered daily via intravenous drip). However, the patient’s condition deteriorated rapidly, necessitating the implementation of invasive mechanical ventilation to maintain adequate oxygenation, along with the administration of vasoactive drugs to sustain stable blood pressure. The antibiotics were further escalated to a five-day course combination of tigecycline (50 mg, administered every 12 hours via intravenous drip) and cefoperazone sodium and sulbactam sodium (3.0 g, administered every eight hours via intravenous drip), because of the presence of *Acinetobacter baumannii* (sensitivity to tigecycline revealed) in the sputum culture. In light of the patient’s persistent fever, the anti-infective treatment was adjusted to increase the dosage of tigecycline (100 mg, administered every 12 hours via intravenous drip), in combination with meropenem(1.0 g, administered every 8 hours via intravenous drip), while keeping the original cefoperazone sodium and sulbactam sodium dosage unchanged, with the aim of improving the antimicrobial effectiveness against *Acinetobacter baumannii*. Despite treatment, the patient’s condition did not improve. Therefore, sivelestat sodium (0.3 g, daily administration via continuous intravenous pump) was administered to mitigate the cytokine storm. Because of the patient’s persistent fever, metagenomic next-generation sequencing (mNGS) of the sputum sample obtained from the intubation tube was performed. The results were as follows: *Haemophilus parainfluenza* (907074 sequence readings); *Acinetobacter baumannii* (118532 sequence readings); *Candida parapsilosis* (25397 sequence readings); *Candida glabrata* (7116 sequence readings); human alphaherpesvirus 1 (HSV1, 21928 sequence readings); human betaherpesvirus 6B (17 sequence readings); and human betaherpesvirus 7 (3 sequence readings). Consequently, a 7 day course of antifungal therapy with caspofungin (50mg, administered daily via intravenous drip) was employed.

**Figure 2:**
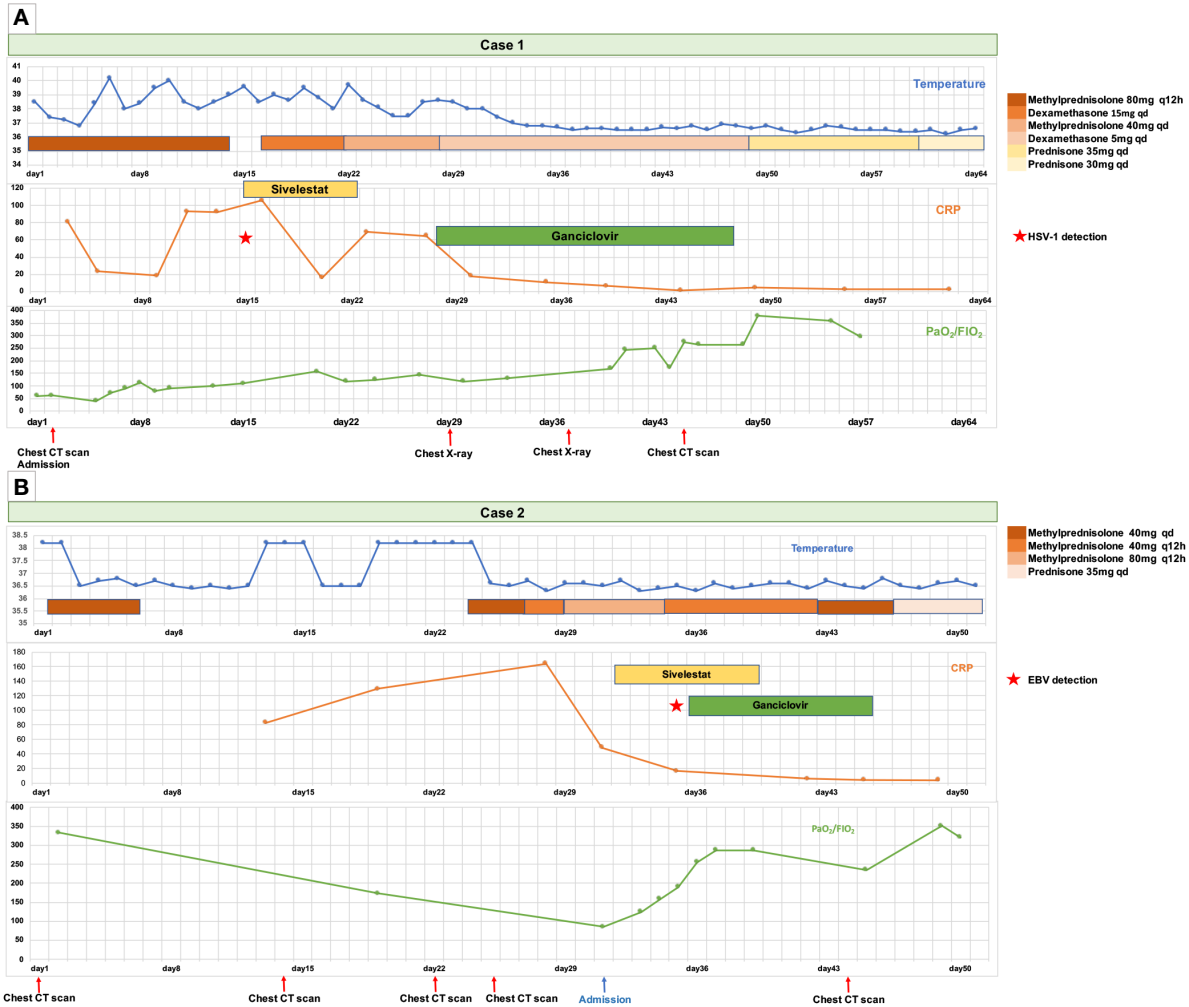
The timeline of disease progression and treatment of the two patients from the onset of symptoms to discharge. **(A)** The timeline of the Case 1 patient. **(B)** The timeline of the Case 2 patient.

Nevertheless, the patient’s condition did not exhibit significant improvement, as evidenced by the persistence of fever, the need for increased ventilator parameters, and the increased diffuse infiltration of both lungs on chest radiography (**Figure 1B**). Consequently, ganciclovir (5 mg/kg, administered every 12 hours via intravenous drip) was administered as a therapeutic intervention against the HHV. Following ganciclovir treatment, the patient experienced significant amelioration, as evidenced by the normalization of body temperature, the reduction of ventilator parameters, and the improvement of the infiltrating chest X-ray images (**Figure 1C**) and infiltrating shadows on chest CT (**Figure 1D**). Following 2 weeks of ganciclovir therapy, mNGS of sputum obtained from the intubation tube did not detect HSV1 but did detect minor levels of HHV 6B and HHV 7. Glucocorticoid administration was gradually tapered. The patient was successfully discharged, and a chest CT examination conducted 40 days post-discharge revealed notable resolution of the pulmonary radiographic findings (**Figure 1E**). The details of the diagnosis and treatment are shown in **Figure 2A**.

### 2.2 Case 2

An 89-year-old Chinese woman presented with recurrent fever and dyspnea (experienced for 1 month prior) and a history of hypertension and a hip replacement 1 year prior. A nasopharyngeal swab PCR tested positive for SARS-CoV-2 RNA on the second day after symptom manifestation. A chest CT revealed multiple bilateral ground-glass opacities (**Figure 1F**). The patient was admitted to a local hospital for a duration of 3 weeks, during which she received a 5-day course of nirmatrelvir/ritonavir, 7 days of intravenous methylprednisolone (40 mg, daily administration via intravenous drip), and antibiotics for 3 weeks (ticarcillin disodium and clavulanate potassium). However, the patient’s symptoms persisted and were characterized by a recurring fever and worsening dyspnea. Subsequent evaluation of the lung CT scan indicated the presence of a newly developed bilateral pleural effusion with a more pronounced extent of lung infiltration compared to the previous assessment (**Figures 1G, H**).

Subsequently, the patient was referred to another hospital, where she underwent an additional three-day regimen of methylprednisolone (40 mg, administered every 12 hours via intravenous drip) therapy in combination with antibiotics (piperacillin sodium tazobactam: 4.5 g, twice daily administration via intravenous drip). The patient’s dyspnea progressively worsened, necessitating high-flow nasal oxygen therapy (HFNT) to sustain adequate oxygen saturation levels. A chest CT revealed additional advancements in lung imaging (**Figure 1I**). Upon admission, the patient presented with acute shortness of breath (respiratory rate: 28 beats/min). Her pulse oxygen saturation level was 94% while receiving 90% FiO_2_ through HFNT. Laboratory examination (**Supplementary Table 1**) showed type I respiratory failure (PaO_2_ 77 mmHg, FiO_2_ 90%), hypoproteinemia (serum albumin 26.8 g/L), increased CRP (49.2 mg/L), increased IL-6 (27.41 pg/mL), serum ferritin increased (850.6 ng/mL), D-dimer increased (1131 μg/L). Quantitative detection of blood EBV DNA was positive, with 2.36 E+04 copies/ml. The patient underwent ganciclovir (5 mg/kg, administered every 12 hours via intravenous drip) treatment, along with an increase in the intravenous methylprednisolone dosage to 80 mg twice daily, and sivelestat sodium for 1 week. The patient’s condition gradually improved, as evidenced by a progressive increase in the oxygenation index, a gradual decrease in the inhaled oxygen concentration of HFNT, and the evident absorption of pulmonary infiltrating shadows on chest CT (**Figure 1J**). Consequently, the methylprednisolone dose was gradually tapered and replaced with oral prednisone tablets. Subsequently, the patient was discharged. Details of the diagnosis and treatment are shown in **Figure 2B**.

## 3 Discussion

The concept of a “multiple hit model” has been introduced as a means to explain the reactivation of persistent pathogens caused by SARS-CoV-2-induced immune dysregulation (Banko et al., 2023). This phenomenon potentially contributes to the advancement and worsening of the disease, necessitating a thorough and comprehensive examination (Banko et al., 2023). According to several studies, individuals infected with SARS-CoV-2 exhibit heightened susceptibility to reactivation of several HHVs, such as VZV, EBV, CMV, HHV-6, and HHV-7 (Ciccarese et al., 2020; Massey et al., 2020; Simonnet et al., 2021; Zubchenko et al., 2022). A meta-analysis indicated that the prevalence of reactivated HHVs in patients with COVID-19 ranges from 3% for HHV-6 reactivation to 41% for EBV reactivation (Banko et al., 2023). Moreover, the likelihood of EBV reactivation was six times higher in critically ill individuals with COVID-19 than in those without COVID-19 (Banko et al., 2023). The infection caused by SARS-CoV-2 has a significant impact on various types of T lymphocytes, specifically CD4+ T cells, CD8+ T cells, and natural killer cells, leading to functional exhaustion and a reduction in their quantity (Xu R. et al., 2020). This subsequent state of immunosuppression may facilitate the reactivation of latent viral infections.

Currently, the relationship between HHV reactivation and long-term COVID-19 has been widely discussed (Peluso et al., 2023; Vojdani et al., 2023). However, the potential correlation between HHV reactivation and exacerbation and an unfavorable COVID-19 prognosis remains unclear. Active HHV infection in patients with COVID-19 has been associated with poor prognoses, such as disease deterioration (Zubchenko et al., 2022) and higher mortality (Xu R. et al., 2020; Meyer et al., 2021; Xie et al., 2021; Carneiro et al., 2022; Gatto et al., 2022). In both our case reports, active HHVs occurred in patients with severe COVID-19. Both conditions improved after treatment with anti-HHV ganciclovir, which might indicate that active HHVs aggravate the disease. The potential risk of poor prognosis resulting from the coinfection of HHVs with SARS-Cov-2 can be attributed to various factors, including the additional strain on the immune system, the potential for immunosuppression, and a heightened hyperinflammatory response (Vigón et al., 2021). EBV can elicit immune dysregulation and stimulate the expression of IL-6 in peripheral blood mononuclear cells through the action of deoxyuridine triphosphate nucleotidohydrolase (dUTPase) *in vitro* (Glaser et al., 2006). Hence, EBV may serve as a supplementary inflammatory stimulus in critically ill patients with COVID-19. Consequently, the potential correlation between HHV reactivation and the exacerbation and advancement of COVID-19 warrants further investigation in future large-scale cohort studies.

Anti-inflammatory glucocorticoids are a fundamental therapeutic approach for the management of severe and critically ill patients with COVID-19. The efficacy of a short course of low-dose dexamethasone at 6 mg/day for a duration of 10 days, or an equivalent dosage of alternative glucocorticoids in severe and critical cases of COVID-19, has garnered validation from various international guidelines and consensus (World Health Organization, ; Bartoletti et al., 2022; National Institutes of Health and Covid-19 Treatment Guidelines Panel, 2023), including the WHO. Nevertheless, despite adhering to the prescribed dosage of glucocorticoids, certain individuals with COVID-19 continue to experience advancement and deterioration of their condition. At present, a consensus on the effectiveness of high-dose corticosteroids in enhancing the prognosis of patients with severe and critical COVID-19 is lacking. Multiple studies have demonstrated that the administration of high-dose corticosteroids can significantly improve the prognosis of patients with COVID-19, particularly those requiring oxygen therapy or mechanical ventilation (Ko et al., 2021; Pinzón et al., 2021; Granholm et al., 2022). However, a study conducted by the COVID STEROID 2 Trial Group revealed that the administration of high-dose corticosteroids (dexamethasone, 12 mg once daily) did not yield any significant reduction in mortality rates at 28 days in patients not requiring life support (Munch et al., 2021). Similarly, a meta-analysis conducted by Tan et al. failed to identify any beneficial effects of high-dose corticosteroid therapy on mortality outcomes (Tan et al., 2022). In each of our cases, corticosteroid administration increased after disease deterioration, suggesting a possible correlation between increased corticosteroid dosage and amelioration of the ailment. Previous research has indicated that high-dose glucocorticoids can be advantageous for patients with COVID-19 who exhibit hyperinflammatory responses (López Zúñiga et al., 2021), whereas such dosages may prove detrimental to individuals without inflammation (Dupuis et al., 2021). Therefore, a potentially more reasonable treatment approach for patients with COVID-19, particularly those with hyperinflammation, could involve an individualized selection of glucocorticoid doses based on their unique characteristics. Glucocorticoids, which are widely available and cost-effective, warrant further investigation to determine whether high-dose administration can effectively ameliorate patient conditions.

In addition to the aforementioned glucocorticoids, sivelestat sodium, a drug that targets anti-neutrophil-related inflammation, demonstrated therapeutic efficacy in both cases. One study indicated that a significant proportion (up to 40%) of hospitalized patients with COVID-19 experience acute respiratory distress syndrome (ARDS), which is the primary driver of mortality among those infected with SARS-CoV-2 (Borges et al., 2020; Michalski et al., 2022). ARDS development may be associated with an unregulated inflammatory response. Pathological observations have revealed that COVID-19 pneumonia can be distinguished by diffuse alveolar damage resulting from the infiltration of macrophages and neutrophils, which closely resembles the pathological manifestations of ARDS (Xu Z. et al., 2020; Bonaventura et al., 2021). Dysregulation of the immune system contributes to the advancement of COVID-19 infection toward a pathogenic state characterized by an increased inflammatory response (Bohn et al., 2020). Neutrophils, which are pivotal effector cells of the innate immune system (Jacobi, 2022), contribute significantly to the development of COVID-19-associated ARDS (Paludan and Mogensen, 2022). The cytokine storm triggered by overactivated neutrophils, neutrophil elastase, and the subsequent release of neutrophil extracellular traps further amplifies the inflammatory response and aggravates lung injury (Al-Kuraishy et al., 2022; Paludan and Mogensen, 2022). Hence, the use of drugs that specifically target neutrophil-associated inflammation is a promising therapeutic intervention for ARDS associated with COVID-19.

To date, the existing reports on hospitalized COVID-19 patients have not extensively addressed the screening tests for HHV infections. In Case 1, the patient demonstrated co-infection with multiple pathogens, as detected through mNGS. It is worth noting that there was an abnormal increase in the abundance of diverse bacteria and fungi, which resulted in the oversight of the possible role of HHVs in disease progression. The crucial turning point in achieving effective patient treatment was the initiation of anti-HHV treatment. The prolonged coexistence of the SARS-CoV-2 with the humans raises concerns about its impact on contemporary society, particularly in the light of an aging population and increasing chronic conditions. The imperative to address the well-being of individuals experiencing severe and critical illness assumes paramount importance. Therefore, further investigation of HHVs activation warrants considerable scholarly attention in the future.

In conclusion, we reported two cases of severe COVID-19 successfully treated for HHV coinfection. These cases demonstrate the need for greater emphasis on the activation of HHVs in patients with severe COVID-19. The initiation of anti-HHV treatment in response to HHV activation under comprehensive treatment with augmentation of glucocorticoid dosage and anti-cytokine storm therapy may improve the prognosis of such patients and enhance the public health outcomes for COVID-19.

## Data availability statement

The raw data supporting the conclusions of this article will be made available by the authors, without undue reservation.

## Ethics statement

Written informed consent was obtained from the individual(s) for the publication of any potentially identifiable images or data included in this article. Written informed consent was obtained from the participant/patient(s) for the publication of this case report.

## Author contributions

YLu: Data curation, Formal analysis, Writing – original draft, Writing – review & editing. CW: Data curation, Writing – review & editing. YW: Data curation, Formal analysis, Writing – review & editing. YC: Conceptualization, Supervision, Writing – original draft, Writing – review & editing. LZ: Formal analysis, Supervision, Writing – review & editing. YLi: Conceptualization, Supervision, Validation, Writing – original draft, Writing – review & editing.
