# Supplementary material for: Case report: Enhancing prognosis in severe COVID-19 through human herpes virus coinfection treatment strategies
Source: Front Cell Infect Microbiol. 2024 Jan 10;13:1320933. doi: 10.3389/fcimb.2023.1320933 (PMC10806028; doi:10.3389/fcimb.2023.1320933)
Supplement: Supplementary file 1 [file Table_1.docx]

Supplementary Material

Case Report: Enhancing Prognosis in Severe COVID-19 through Human Herpes Virus Coinfection Treatment Strategies

Ye Lu, Cuihong Wang, Yuan Wang, Li Zhao, Yu Chen, Yu Li *

*** Correspondence:** Yu Li: sundy_liyu@163.com

# Supplementary Table 1

## Supplement Table 1 Laboratory findings of the two patients after admission

|  | Case 1 | Case 2 | Reference range |
| --- | --- | --- | --- |
| **Arterial blood gas analysis** |  |  |  |
| PH | 7.50 | 7.48 | 7.35 – 7.45 |
| PaO_2_ | 60 (mask oxygen therapy with 15L/min) | 77 (HFNC therapy with FiO_2_ of 90%) | 75 - 100 |
| PaCO_2_ | 44 | 48 | 35 - 45 |
| **Blood routine** |  |  |  |
| white blood cell count,10^9/L | 6.41 | 5.97 | 3.5 – 9.5 |
| Lymphocyte count，10^9/L | 0.3 | 0.3 | 1.1 – 2.7 |
| Hemoglobin, g/L | 146 | 94 | 130 – 172 |
| Platelet count, 10^9/L | 94 | 245 | 135 – 350 |
| **Blood biochemical test** |  |  |  |
| Serum albumin, g/L | 34.5 | 26.8 | 35 – 53 |
| ALT, U/L | 101 | 14 | 0 – 40 |
| AST, U/L | 133 | 11 | 5 – 34 |
| Serum creatinine, μmol/L | 65.5 | 10.21 | 59 – 104 |
| Blood urea nitrogen, mmol/L | 6.45 | 10.21 | 3 – 9.2 |
| Serum potassium, mmol/L | 3.56 | 4.28 | 3.5 – 5.5 |
| Serum sodium, mmol/L | 140 | 136 | 136 – 145 |
| D-dimer, ug/L | 679 | 1131 | 0 – 252 |
| Ferritin, ng/mL | 1275.2 | 923.1 | 11 – 336.2 |
| IL-6, pg/mL | 7.16 | 27.41 | ≤ 7 |
| C-reactive protein, mg/L | 134.4 |  | 0-6.0 |
| Lactic dehydrogenase, U/L | 824 | 268 |  |
| **Serum viral antibody** |  |  |  |
| HSV (1+2) IgM | - | - | - |
| HSV(I+2) IgG | + | + | - |
| CMV-IgM | NA | - | - |
| CMV-IgG | NA | + | - |
| EBV-IgM | NA | - | - |
| EB-EA-IgG | NA | + | - |
| EB-NA-IgG | NA | + | - |
| EB-VCA-IgG | NA | + | - |
| Serum EBV-DNA, IU/mL | _ | 2.36E+04 |  |
| Serum CMV-DNA, IU/mL | _ | _ |  |
| Sputum culture | Acinetobacter baumannii | _ |  |
| **mNGS, sequence readings** |  | **NA** |  |
| HSV-1 | 21928 |  |  |
| HHV-6B | 17 |  |  |
| HHV-7 | 3 |  |  |
| Haemophilus parainfluenzase | 907074 |  |  |
| Acinetobacter baumannii | 118532 |  |  |
| Candida parapsilosis | 25397 |  |  |
| Candida glabrata | 7116 |  |  |

HFNC, high-flow nasal cannula; FiO2, fraction of inspire oxygen; PH, Pondus Hydrogenii; PaO2, arterial partial pressure of oxygen; PaCO2, arterial partial pressure of carbon dioxide; ALT, Alanine aminotransferase; AST, Aspartate aminotransferase; IL-6, interleukin-6; EBV, Epstein–Barr virus; CMV, cytomegalovirus; HSV-1, herpes simplex virus type 1; HHV-6B, human betaherpesvirus 6B; HHV-7, human betaherpesvirus 7;
